# Influencing of coal industry related airborne particulate matter on ocular surface tear film injury and inflammatory factor expression in Sprague-Dawley rats

**DOI:** 10.1515/biol-2025-1094

**Published:** 2025-10-15

**Authors:** Ran Zhu, Quan Zou, Hanbing Wei, Jintuo Zhu, Xinjian He

**Affiliations:** School of Safety, China University of Mining and Technology, No. 1, University Road, Tongshan District, Xuzhou, 221116, Jiangsu, P. R. China; Center of Refraction, The Affiliated Xuzhou Municipal Hospital of Xuzhou Medicine University, Xuzhou, 221000, Jiangsu, P. R. China

**Keywords:** airborne particulate matter, ocular surface, tear film, IL-6, IL-17, TNF-α

## Abstract

To investigate the effect of coal industry-related airborne particulate matter (PM) on ocular surface tear film injury and inflammatory factor expression. Male Sprague-Dawley (SD) rats were randomly divided into the treatment group and the normal control group, with five rats in each group. A dust chamber was used to simulate the air contamination conditions associated with the coal industry. Tear secretion, tear-film breakup time (BUT), conjunctival congestion score, and relative expression levels of tear inflammatory factors, including interleukin (IL)-6, IL-17, and tumor necrosis factor (TNF)-α, were compared between the treatment group and the normal control group. After 4 weeks’ exposure, tear secretion (2.64 ± 0.57 mm vs 5.42 ± 0.28 mm), BUT (4.23 ± 0.47 s vs 6.15 ± 0.36 s), and conjunctival congestion score [2 (2, 3) vs 0 (0, 1)] were significantly different between the treatment group and the control group (all *P* < 0.05), and hematoxylin-eosin stain showed that the number of goblet cells decreased in the treatment group. In addition, the relative expression levels of IL-6, IL-17, and TNF-α in tears of the treatment group were significantly different from those of the normal control group (all *P* < 0.05). Coal industry-related airborne PM exposure can damage tear film function and increase relative expression levels of tear inflammatory factors in SD rats.

## 1 Introduction

Coal has been the main fossil fuel for a long time, and the proportion of coal in the structure of primary energy production and consumption exceeds 70% in China [1]. According to the “adhering to the energy strategy of taking coal as the main body, electric power as the center, oil and gas and new energy for comprehensive development,” the coal industry will remain China’s predominant energy industry for a long time to come [2]. However, different degrees of air pollution can result from the process of coal mining, drilling, blasting, trucking and transportation, rubble dumping, and/or other operations, which has become an important public health problem affecting human health [3].

Therein, particulate matter (PM) causes damage not only to the respiratory system but also to the ocular surface. Ambient PM increased the rate of ophthalmology visits due to ocular surface irritation and allergies, and this association increased in magnitude with higher ambient PM concentrations [4]. PM has been primarily observed to induce allergic reactions, inflammatory responses, oxidative stress, mitochondrial impairment, DNA damage, and inhibit the proliferation and migration of ocular surface cells [5,6,7]. These effects ultimately result in impaired wound healing and ocular surface damage. Recent study data supported the association between exposure to PM and the development of ocular pathologies such as retinal atherosclerosis, dry eye (DED) syndrome, and glaucoma [8,9]. DED is a multifactorial disease of the ocular surface characterized by a loss of homeostasis of the tear film and accompanied by ocular symptoms [10]. Also, PM can alter tear composition that may contribute to ocular diseases such as DED, blepharitis, keratitis, conjunctivitis, pterygium, and limbal stem cell deficiency [5]. Dang et al. [11] found that air pollution during the operation, such as PM pollution, changes tear film function, including eye redness index and noninvasive tear film break-up times. Nevertheless, the exact effect of air pollution related to the coal industry, especially PM exposure, on tear film function is not clear.

Since such a series of issues cannot be ignored, the potential mechanism that PM affecting ocular surface tear film injury needs to be revealed. Hence, in this study, a “dust chamber” was utilized to simulate air contamination conditions to investigate the effects of PM on ocular surface tear film and different inflammatory factors in tear in Sprague-Dawley (SD) rats, and provide some basic information for the understanding of the interaction between air contamination associated with coal and the health status.

## 2 Methods

### 2.1 Animals

Healthy male SD rats (weighting 250–350 g) were randomly divided into the treatment group and the normal control group according to the random number table, with five rats in each group. The rats were purchased from the Vitonglihua (Beijing) Biotechnology Co., Ltd., and raised in the Animal Experimental Center of Xvzhou Medical University. Before the experiment, slit-lamp microscopy was used to exclude rats with conjunctivitis, keratitis, and other ocular surface-related diseases. The SD rats were raised uniformly in a standard environment during the experiment. The handling and treatment of laboratory animals were carried out in accordance with the guidelines of the Animal Experimental Center of Xuzhou Medical University.


**Ethical approval:** The research related to animal use has been complied with all the relevant national regulations and institutional policies for the care and use of animals and has been approved by the Animal Ethics Committee of Xuzhou Medical University (No. 200403T004).

### 2.2 Experimental design

**Figure 1:**
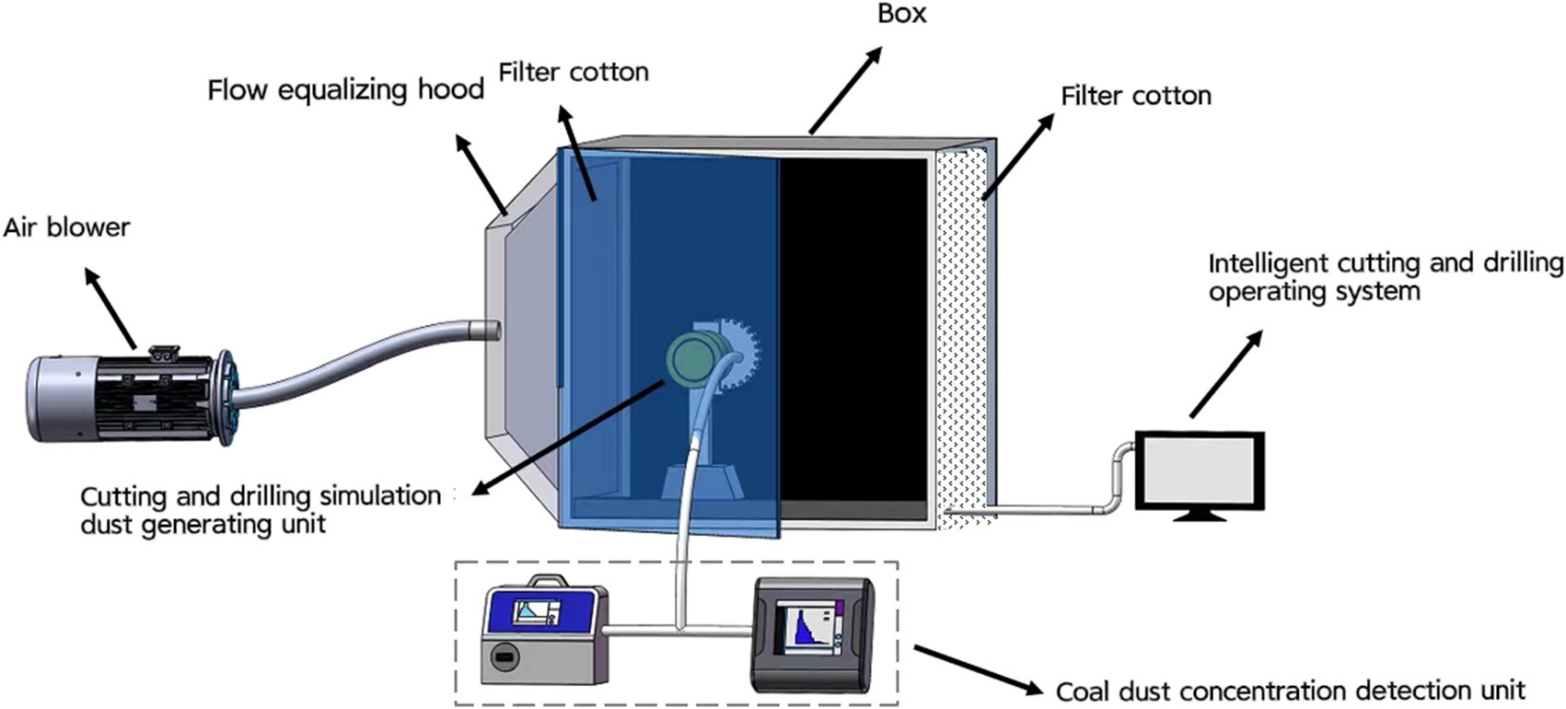
The schematic diagram of dust chamber used to simulate the air contamination condition.

A dust chamber (L-RD0600B) was used to simulate the air contamination condition to treat the SD rats, which was purchased from Jiufang Electronics Co., Ltd (Guangzhou, China). The experimental anthracite powder is used to produce PMs and was ground into PMs with different grain sizes, including PM 2.5 and PM 10. The dust accumulation in the cutting disk, drill bit, and chamber has been cleaned ahead of the experiment to reduce its interference to the dust production detector. Then, we opened the fan and adjusted the air volume to form a continuous and stable clean airflow inside the chamber. According to the guidance of the Coal Mine Safety Regulations on coalface ventilation, the ventilation speed was set at 0.3 m/s. The exposure concentration maintained dynamic stability. The coal body was cut and drilled through a roller and drill bit controlled by the dust-producing unit, and the monitoring started when the PM concentration became stable, with the monitoring point arranged on the side of the roller and drill bit, and the monitoring time is 1 min (Figure 1). Based on the pre-experiment that showed the PM concentration tended to be stable after 2 min of cutting and drilling, and therefore, time for monitoring was set at 2 min after the coal body cut and drilled. In addition, the control variate method was used for parameters setting, and the experiment was repeated three times under the same combination condition to reduce the deviation, with the experiment order being completely random.

### 2.3 Tear secretion measurement

The phenol red cotton thread test was used to accurately evaluate tear secretion in SD rats, in which the filament cotton thread soaked by phenol red solution was initially yellow and turned to red after touching tear. The volume of tear secretion was quantified through measuring the length of the red part on the cotton thread. To track the change in tear secretion after dust exposure, the measurement was performed 1 day before exposure and 4 weeks after exposure.

In the experimental procedure, we first fixed the head of SD rats and lightly spread the palpebra inferior to fully reveal the conjunctival sac. Next, the phenol red cotton thread pre-bent to 2 mm was placed one-third of the palpebra inferior conjunctival sac, and it was removed after remaining for 20 s. The length of the red part on the cotton thread (in mm) was then accurately measured. Operations with slit-lamp were carried out by the same researcher person in the constant darkroom environment during the whole process to ensure the accuracy of the results. Three repeated measurements were taken for each eye, and the average value was taken as the final data.

### 2.4 Breakup time (BUT) determination and conjunctival congestion assessment

The 1% sodium fluorescein solution was accurately dropped into the conjunctival sac of SD rats using a pipette, and after three times artificially assisted blinking, keeping the eyelids open. The filter of slit-lamp was adjusted to cobalt-blue light mode to observe changes in the tear film of SD rats. Timekeeping started at the last blink, and the first time point of tear-film breakup was recorded, namely, the time of the first dark spots appear. Measurements were repeated three times and the mean value was used as the BUT. In addition, the conjunctival congestion was assessed according to the study conducted by Efron [12], which was divided into four grades with scores of 0 (normal) to 4 (severe). Specifically, score 0 represents white bulbar conjunctiva, a major blood vessel, clear and clear cornea, and absence of congestion; score 1 represents slight congestive conjunctival, major blood vessels, marginal corneal limbus congestion, but no significant conjunctival congestion; score 2 represents that the conjunctival congestion is red, the corneal limbus is slightly congested, the ciliary is slightly congested, and there was mild congestion in the conjunctiva and limbus; score 3 represents increased conjunctival congestion, moderate corneal limbus congestion, and moderate ciliary congestion, and the conjunctiva and limbus of cornea showed obvious congestion, but it was not severe; and score 4 represents severe conjunctival hyperemia, severe limbal hyperemia, and major blood vessels clear, and the conjunctiva is extensively congested, affecting the transparency of the cornea.

### 2.5 Tissue extraction and fixation

After the rats were killed, skin and musculature were scissors along the orbital margin, the eyeball and appendages were separated close to the orbital bone, and the optic nerve was cut to completely obtain eyeball and eyelid skin with appendages, with the conjunctival sac maintained intact. The tissues were washed in pre-cooling phosphate-buffered solution (PBS) buffer to scour off the blood; after trimming the excess muscle and fat tissue under a microscope, the tissues were fixed overnight in 4% paraformaldehyde solution. The fixed tissue was rinsed with running water for several hours and cut along the optic nerve by a micro scissor, and posterior scleral tissue was trimmed. After peeling the crystalline lens, the tissue was washed in PBS buffer again. The trimmed eyeball and appendages were placed in a dehydration box, and then the dehydration box was placed in an automatic dehydrator for gradient alcohol dehydration.

Before preparing the wax-impregnated tissue blocks for embedding, we first preheat the fixed splint in an oven at 65°C. A small amount of wax was poured into the mold, and the wax-impregnated tissue blocks were gently placed into the mold using tweezers, ensuring that the axis of the eyeball was parallel to the long side of the embedding frame. Then cover the preheated fixed splint and pour the remaining wax liquid, so that the fixed plate could be completely immersed in the wax liquid. After that, the whole mold was moved to a frozen table at −20°C. After the wax liquid was cooled and solidified, the wax blocks were removed from the mold for preparation of paraffin sections.

### 2.6 HE staining

Paraffin sections were rinsed with 1× PBS buffer for three times, 5 min each time, and put into the hematoxylin staining solution for 3 min. After staining, the floating color was washed with running water after, and the sections were differentiated by 1% hydrochloric alcohol for 2 s. Then the sections were rinsed with water for 10–20 min to return to blue, and after washing with distilled water for a few seconds, they were stained in the Eosin staining solution for 1 min. The slides were, respectively, dehydrated in 85, 95, and 100% ethanol for 2 min in turn, and sealed with neutral gum. After drying at room temperature, the slides were observed and photographed by a light microscope, where the nuclei were in blue and the cytoplasm was in red.

### 2.7 Expression of inflammatory factors

Tear of SD rats were absorbed by the capillary glass tube, and the supernatant solution was collected after centrifugation. Enzyme-linked immunosorbent assay was utilized to evaluate the relative expression levels of interleukin (IL)-6, IL-17, and tumor necrosis factor (TNF)-α. Samples, standards, and biotin-labeled antibody horseradish peroxidase (HRP) conjugate were added into microwell plate precoated with rat IL-trapping antibodies in sequence. After incubation and washing, the TMB substrate was used for color reaction, in which the TMB was converted to blue under the catalysis of HRP and transformed into the final yellow color under the action of acid. The depth of the color was positively correlated with the rat IL in the sample. Absorbance (with the unit of optical density value) was measured by the enzyme-labeled instrument at 450 nm wavelength, and the expression levels of inflammatory factors between the normal control group and the treatment group were calculated.

### 2.8 Image processing and statistical analysis

SPSS 26.0 software was used for statistical analyses. Measurement data were expressed in the form of mean ± standard deviation (SD) to ensure the accuracy and intuitiveness. The GraphPad Prism 10 software was employed for analyses and plotting. One-way analysis of variance test was used for comparison between the treatment group and the normal control group, and two-sided *P* < 0.05 represented the difference was statistically significant.

## 3 Results

### 3.1 Ocular surface tear film injury in SD rats

After 4 weeks of dust exposure, tear secretions of SD rats in the treatment group and the normal control group were, respectively, 2.64 ± 0.57 mm and 5.42 ± 0.28 mm. Compared with the normal control, SD rats exposed to the simulative airborne PM had significantly higher levels of tear secretion (*F* = 5.251, *P* < 0.001). The BUT of rats was also significantly shorter in the treatment group (4.23 ± 0.47 s) than that in the normal control group (6.15 ± 0.36 s), with *F* = 6.351 and *P* < 0.001. Also, the conjunctival congestion scores in the treatment group and the normal control group were 2 (2, 3) and 0 (0, 1), respectively.

### 3.2 Ocular surface tissue morphology and expression of inflammatory factors

**Figure 2:**
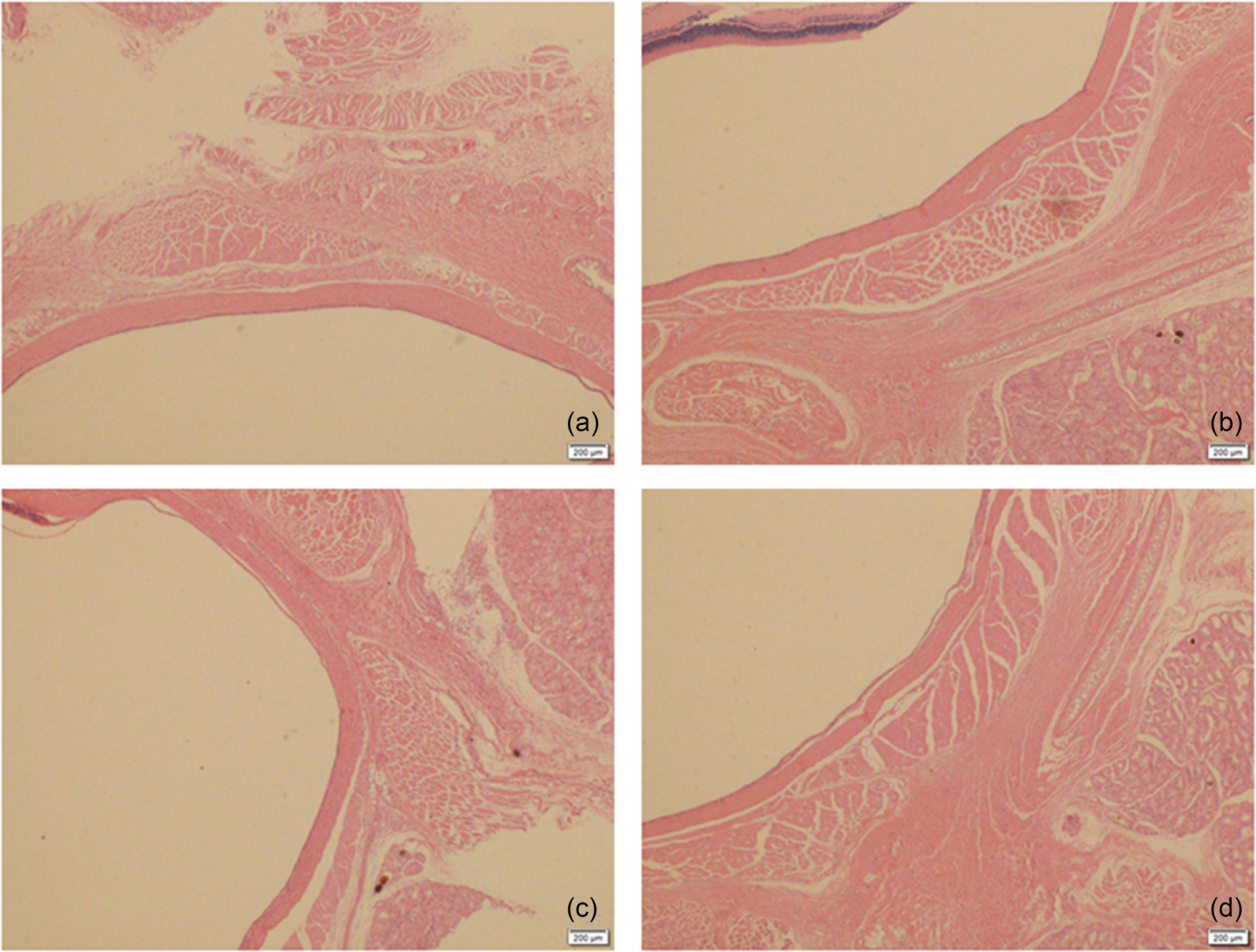
HE staining of ocular surface tissues in rats. (a) and (b) normal control group, and (c) and (d) treatment group.

**Figure 3:**
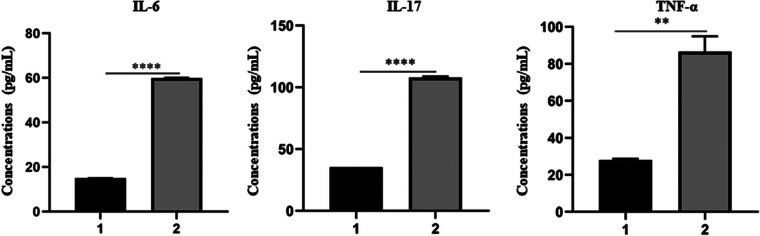
Protein expression levels of IL-6, IL-17, and TNF-α in tear of rats between the treatment group and the normal control group.

Figure 2 shows that HE staining showed that the nucleus of goblet cells of ocular surface tissues in rats in the normal control group was plump, normal in shape and neatly arranged (a and b). Differently, the structure of some goblet cells was obviously destroyed, the density decreased, the number of goblet cells decreased, and the arrangement was chaotic in the treatment group (c and d). In addition, Figure 3 shows protein expression levels of IL-6, IL-17, and TNF-α in tear of rats between the treatment group and the normal control group. The results suggested that relative expression levels of IL-6, IL-17, and TNF-α in tear were all significantly higher in rats exposed to PMs than those in normal controls (all *P* < 0.05).

## 4 Discussion

This study used a dust chamber to simulate the air contamination condition at the mine site to treat SD rats, and the results showed that SD rats in the exposure group have significantly higher levels of tear secretion, shorter BUT, and higher conjunctival congestion scores, compared to normal controls. The structure of some goblet cells was obviously destroyed, the density decreased, the number of goblet cells decreased, and the arrangement was chaotic in the treatment group. Also, the relative expression levels of IL-6, IL-17, and TNF-α in tear were all significantly higher in rats exposed to PMs than those in normal controls.

Due to the strong diffusivity and volatility of PM, the mechanical damage of PM particles to human health has attracted widespread attention, but the impact on ocular surface diseases has been rarely reported. In recent years, taking China as an example, studies included the eye data of 387 patients from 11 hospitals in 5 provinces (namely, Beijing, Hebei, Heilongjiang, Anhui, and Inner Mongolia) and found that increased PM exposure could cause eye discomfort and tear film instability damage [13,14]. A follow-up study was conducted on 50 healthy adults living in Chiang Mai, Thailand, and the results showed that exposure to PM 2.5 significantly raised the likelihood of ocular redness, watering, and dryness; and lymphocyte counts were also positively correlated with redness, watering, and dryness during high PM 2.5 exposure [15]. Another study in an experimental allergic eye disease mouse model demonstrated that exposure to PM significantly exacerbates ocular allergy, evidenced by increased eye-lid edema, mast cell degranulation, inflammatory cytokines, cell proliferation, and serum IgE, polymorphonuclear leukocytes, and apoptosis and reduced goblet cells [6]. As mentioned earlier, animal models resulted in phenotypes reminiscent of that of DED, presenting with reduced tear volumes and ocular surface damage [9]. The prevalence of PM-induced ocular surface injury and DED is 5–50% over the world, and 21–30% in China [16]. Hao et al. [17] conducted a prospective multicenter cohort study, and 387 DED participants were recruited from five provinces in China and suggested that increased PM 2.5 and PM 10 exposures caused ocular discomfort and damage with tear film instability. Air pollution, including airborne PMs, can result from the process of coal operations, as well as PM in different concentrations and particle sizes, and multiple micro-physical and chemical parameters have different effects on the ocular surface. Therefore, the current study used anthracite particles to investigate the effect of airborne PMs with different particle sizes on ocular tear film, which may provide some information for the prevention of DED in coal miners and residents in coal industrial areas.

In recent years, due to changes in work and lifestyle, aggravation of environmental pollution, and other factors, the prevalence of DED has further increased [18,19]. Differing from other tissues or organs, the ocular surface is isolated from the pollutants in the environment by a thin layer of tear film. Long-term exposure to the polluted environment makes the ocular surface more susceptible to the pollution than other organs or tissues, and DED has also become the most common eye disease for a long time. The ocular surface microenvironment is composed of different tissues, cells, extracellular matrix, and other complex components at multiple levels, including eyelid, cornea, conjunctiva, meibomian gland, lacrimal gland, tear film, immune system, innervation system, endocrine regulation system, blood vessel and lymphatic system, and microbial community. These components are interrelated and influence each other to maintain a stable and healthy ocular surface. Therefore, the maintenance of microenvironment homeostasis of ocular surface requires the comprehensive action of many factors to achieve a balanced state. Different pathogenic factors, such as abnormal quality and quantity of tear or dynamics, may not only lead to tear film instability but also affect the larger scope of the ocular surface tissue microenvironment. When one or more tissues, cells, extracellular matrix, and other components change, it often causes a chain reaction, resulting in other microenvironment components to change; if the degree of change exceeds the compensatory ability of the body, it will eventually lead to the imbalance of the homeostasis of the ocular surface microenvironment. Tear film covering the outer layer of the ocular surface is an important part of maintaining the health of the ocular surface and optical characteristics and preventing microbial invasion. Tear film, cornea, and intraocular dioptric media are also important links affecting retinal imaging, among which tear film is the first dioptric media on the ocular surface [20]. Therefore, the stability of tear film is a crucial adjective to ensure the quality of retinal imaging.

The stability of the tear film must be guaranteed by the normal composition of the tear and the normal movement and inextricable nerves of the eyelid. Evidences have shown that eye injury can be manifested as conscious symptoms such as eye friction, pain, photophobia, tearing, and blurred vision. High concentrations and prolonged exposure to air pollution can lead to acute keratitis, peeling of large sections of corneal epithelium, and irreparable corneal epithelium within 24 h [21,22]. Also, experts agreed that chronic ocular surface diseases are the instability of tear film or the imbalance of ocular surface microenvironment caused by abnormal tear quality, quantity, and dynamics, which may be accompanied by ocular surface inflammatory response, tissue damage, and nerve abnormalities, resulting in a variety of ocular discomfort symptoms and/or visual dysfunction [23,24].

**Figure 4:**
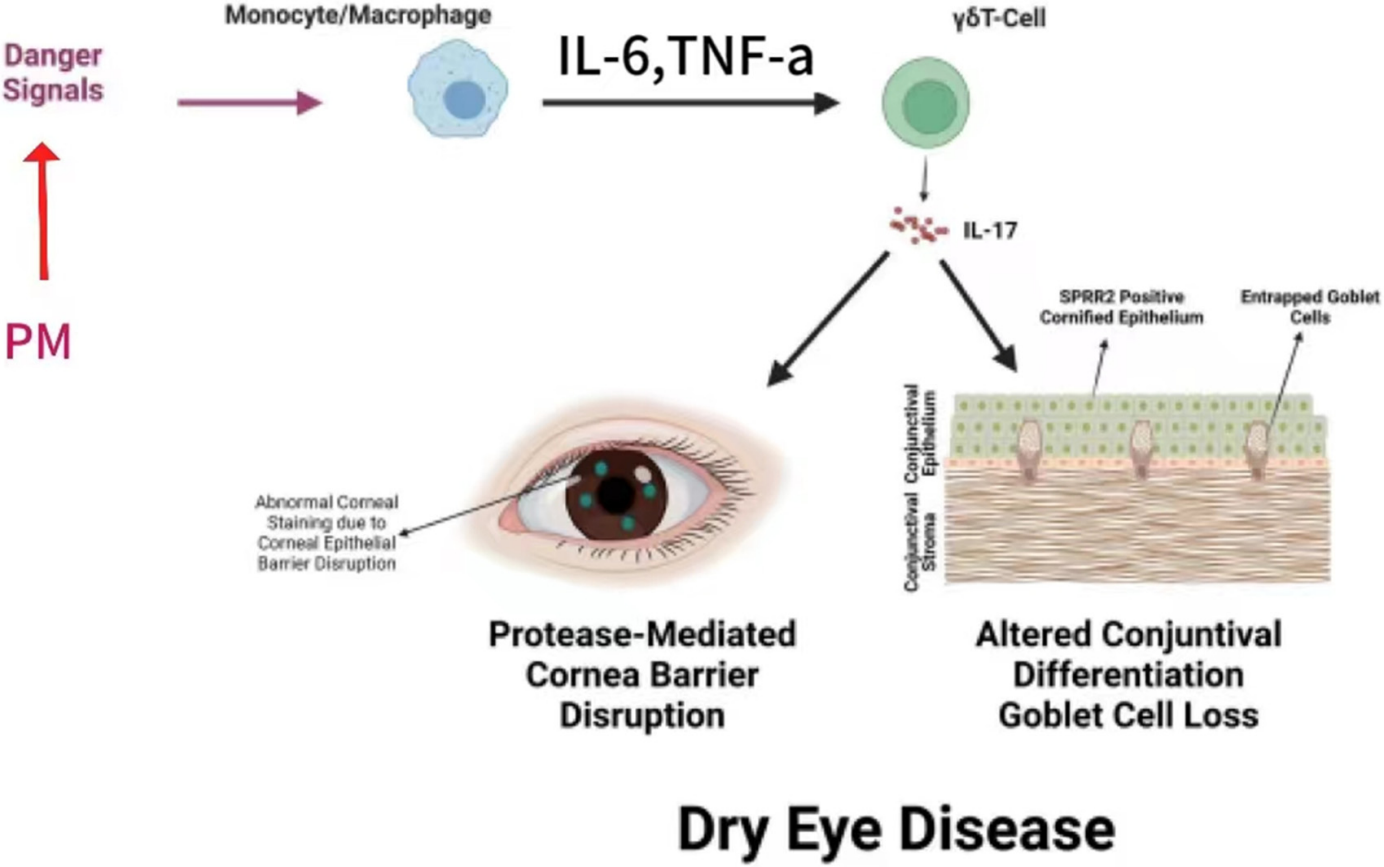
Potential mechanism that influencing of PM on DED.

Researchers have found that the underground miners have lower tear secretion and shorter BUT than that of other workers who are not exposed to PM [25]. Therefore, PM exposure plays an important role in the occurrence and development of DED. The harm caused by PM to the human body varies due to different diameters, particle-size distribution, chemical composition, sources, and formation conditions. Among them, PM 10 and PM 2.5 have especially raised the most concern. In the present study, PM in the dust chamber was recorded according to their aerodynamic size, including (1) the total suspended PM with grain diameter ≤100 μm; (2) inhalable particle (generally known as PM 10) with grain diameter ≤10 μm, which can enter the human respiratory, circulation and other systems; (3) fine particles (generally known as PM 2.5) with grain diameter ≤2.5 μm; and (4) ultrafine PM (generally known as PM 0.1) with grain diameter ≤0.1 μm. At present, PM as the main component of haze is mainly emitted from anthropogenic combustion, which may have a negative impact on human health [26]. Long-term exposure to PM pollution is more likely to affect ocular surface than the respiratory mucosa [27,28]. In addition to the mechanical damage of PM, the cause of DED may be also associated with the toxicity of PM, and however, the specific mechanism remains to be further clarified (Figure 4). Our results suggested that after 4 weeks of exposure to the dust chamber, the expression level of IL-6 in the conjunctival tissue of rats continued to increase, inducing an inflammatory response in the ocular surface environment.

DED is an inflammatory disease that shares many features with autoimmune diseases. The stress response of the ocular surface to environmental factors, infection, endogenous stress, antigen, and so on is considered to be the trigger mechanism of DED. We observed that compared with the normal control group, the BUT of SD rats in the dust group was significantly decreased, and the expression levels of tear inflammatory factors (including IL-6, IL-17, and TNF-α) were significantly increased. In both meibomian gland and meibomian gland dysfunction rat models of DED patients, elevated levels of pro-inflammatory cytokines (TNF-a), which are immunomodulators secreted by different types of cells and play an important role in cell signaling, such as pro-inflammatory or anti-inflammatory effects, were similarly detected [29]. TNF-a is a pleiotropic cytokine with a wide range of biological activities, and as an important pro-inflammatory factor, it can activate and affect a variety of cell types, including immune cells, endothelial cells, and fibroblasts, triggering a series of biological responses. The powerful pro-inflammatory and co-stimulatory effects make these inflammatory factors play pivotal roles in inflammatory response and immune regulation. They also play a key role in the defense against pathogen invasion, and when the body is attacked by external pathogens, they can respond quickly and coordinate immune cells and other related cells to jointly resist the invasion of pathogens.

Inflammation is a common feature, and both a cause and a consequence of different types of DED. Pro-inflammatory cytokines, chemokines, and matrix metalloproteinases lead to the expansion of autoreactive T cells, thus infiltrating the ocular surface and lacrimal gland, causing an increase in the number of eyelid blink, invasion of a large number of lymphocytes in the lacrimal gland and ocular surface tissue, and the release of inflammatory factors leading to immune-related inflammation, which can impair nerve conduction of normal tear secretion, and thus affect the quality and quantity of tear secretion, and form a vicious cycle. Our findings indicated a possible pathway that anti-inflammation could be a possible measure to prevent PM from causing damage to ocular surface tear film from the root and cut off the cascade reaction of subsequent tear inflammatory factor expression leading to DED. Based on the results of the current study and previous studies, it was speculated that PM exposure may damage the tear film function by damaging epithelial cells, destroying conjunctival goblet cells, inducing oxidative damage of ocular surface tissues, or activating inflammatory response [30,31]. Inflammation is an important factor that leads not only to the vicious cycle of DED but also to the chronic DED, and therefore, controlling inflammation is the basis for the prevention and treatment of chronic DED. There is evidence that chronic inflammation in DED causes changes in epithelial cell morphology and function, and in the case of prolonged stimulation, the expression of other molecules (such as intercellular adhesion molecule-1) may trigger the activation of adaptive immune pathways, allowing lymphocytes to migrate to the conjunctiva, and triggering chronic immune-mediated inflammatory responses. Controlling and reducing ocular surface inflammation, which can result from epithelial damage and environmental stressors, may be a key component of all treatment options.

Furthermore, the damage of ocular tear film and its possible mechanism were detected in rats exposed to coal dust particles in a simulated dust chamber in this research, which may provide information for the prevention of ocular surface diseases such as DED under the exposure of air particles related to the coal industry to a certain extent. However, since the dust chamber that simulated the air contamination condition could only hold five rats at one time, the sample size was constant, and such a small sample size may raise concerns about statistical power. Drug intervention after the establishment of the DED eye model is planned in the next research plan, and further, there is a need to improve the clinical studies on both animals and DED patients.

## 5 Conclusion

PM not only causes damage to the respiratory system but also causes tear film injury on the ocular surface, changes in the expression of inflammatory factors, and may further induce DED. With the increasingly serious air pollution, anti-inflammation could be applied to prevent PM from causing damage to the ocular surface tear film and cut off the cascade of subsequent tear inflammatory factor expression, which may become a new way to prevent DED.
